# Integrated RNA-seq and sRNA-seq analysis identifies novel nitrate-responsive genes in *Arabidopsis thaliana* roots

**DOI:** 10.1186/1471-2164-14-701

**Published:** 2013-10-11

**Authors:** Elena A Vidal, Tomás C Moyano, Gabriel Krouk, Manpreet S Katari, Milos Tanurdzic, W Richard McCombie, Gloria M Coruzzi, Rodrigo A Gutiérrez

**Affiliations:** 1 FONDAP Center for Genome Regulation. Millennium Nucleus Center for Plant Functional Genomics, Departamento de Genética Molecular y Microbiología, Facultad de Ciencias Biológicas, Pontificia Universidad Católica de Chile, Santiago 8331010, Chile; 2 Biochimie et Physiologie Moléculaire des Plantes, UMR 5004 CNRS/INRA/SupAgro-M/UM2, Institut de Biologie Intégrative des Plantes-Claude Grignon, Montpellier, France; 3 Center for Genomics and Systems Biology, Department of Biology, New York University, New York, NY 10003, USA; 4 Cold Spring Harbor Laboratory, New York, USA; 5 Present address: School of Biological Sciences, The University of Queensland, St Lucia, Australia

**Keywords:** Arabidopsis, Nitrate, RNA-seq, Roots, MicroRNA, Transcriptomics

## Abstract

**Background:**

Nitrate and other nitrogen metabolites can act as signals that regulate global gene expression in plants. Adaptive changes in plant morphology and physiology triggered by changes in nitrate availability are partly explained by these changes in gene expression. Despite several genome-wide efforts to identify nitrate-regulated genes, no comprehensive study of the Arabidopsis root transcriptome under contrasting nitrate conditions has been carried out.

**Results:**

In this work, we employed the Illumina high throughput sequencing technology to perform an integrated analysis of the poly-A + enriched and the small RNA fractions of the *Arabidopsis thaliana* root transcriptome in response to nitrate treatments. Our sequencing strategy identified new nitrate-regulated genes including 40 genes not represented in the ATH1 Affymetrix GeneChip, a novel nitrate-responsive antisense transcript and a new nitrate responsive miRNA/TARGET module consisting of a novel microRNA, miR5640 and its target, AtPPC3.

**Conclusions:**

Sequencing of small RNAs and mRNAs uncovered new genes, and enabled us to develop new hypotheses for nitrate regulation and coordination of carbon and nitrogen metabolism.

## Background

Nitrogen (N) is an essential macronutrient and a key factor controlling plant growth and development. Nitrate is the main form of N available in agricultural soils [1-3]. Nitrate is taken up by the cell by specific nitrate transporters and is reduced to nitrite in the cytoplasm by nitrate reductase. Nitrite is reduced to ammonium in the plastid by nitrite reductase and is incorporated into amino acids by the glutamate synthase/glutamine synthetase cycle (GS/GOGAT cycle). Nitrate metabolism is tightly coordinated with carbon metabolism, since carbon skeletons in the form of 2-oxoglutarate are required for ammonium assimilation [1,4]. One of the most striking examples of plant plasticity in response to changing environmental conditions is root system architecture modulation by changes in nitrate availability (for reviews see [5-7]). In order to identify molecular mechanisms underlying these changes, transcriptomics analyses of the nitrate response of Arabidopsis have been performed, most of them utilizing the Affymetrix ATH1 GeneChip. Analyses with the ATH1 chip showed that nitrate is able to regulate more than 2,000 genes in roots, some of them responding as fast as 3–6 minutes after nitrate exposure [8] and including genes involved in nitrate transport, reduction and assimilation, hormone signaling pathways, transcription factors, kinases and phosphatases, among others [8-12]. However, a detailed view of the transcriptomics changes triggered by nitrate has been limited by the representation of genes in the ATH1 microarray. ATH1 contains probe sets representing approximately 21,000 genes allowing for the detection of only 71% of the genes annotated in the Arabidopsis genome v.10. Moreover, these probes do not include important regulatory elements of the genome such as small (sRNAs).

High-throughput sequencing technologies allow for quantitative determination of RNA levels and RNA sequencing (RNA-seq) is becoming the technology of choice to investigate the transcriptome. RNA-seq offers several advantages over hybridization-based techniques like microarrays [13-18]. RNA-seq is not limited to detection of transcripts that correspond to annotated genes, thus it allows for identification of new genes. RNA sequencing can also be utilized to analyze the sRNA component of the transcriptome when libraries are prepared from low-molecular weight RNA fractions [19-24]. microRNAs (miRNAs), short interfering RNAs (siRNAs) and other types of sRNAs have been shown to play important roles in a broad range of biological processes, such as plant development and response to biotic and abiotic stresses [25-29], including plant responses to various nutrients [30-37].

In plants, the sRNA transcriptome is primarily composed of 23–24 nt siRNAs and 21–22 nt miRNAs [36,38,39]. Since miRNA precursors have distinctive secondary structures, many bioinformatics programs have been developed to predict new miRNAs based on sequencing of a sRNA in a library and inspection of the genome sequence containing this sequence for putative miRNA precursors [40-42]. Combination of deep sequencing approaches and bioinformatics predictions have identified 19,724 miRNAs related sequences across different phyla out of which 266 correspond to Arabidopsis miRNAs in miRBase v.17 [43].

miRNA regulation of nitrate-responsive genes has been shown to be a key mechanism of plant responses coordinating nitrate availability and root developmental responses. miR167 is down-regulated by nitrate treatments in pericycle cells and this leads to an induction of its target, the auxin response factor ARF8 [44]. Regulation of ARF8 by miR167 causes a change in the ratio of initiating and emerging lateral roots in response to nitrate [44]. Another nitrate regulatory module, consisting of miR393 and the AFB3 auxin receptor has been shown to control root system architecture in response to external and internal nitrate availability [37]. Microarray analysis suggests that other miRNAs can be involved in root responses to nitrate, since several miRNA targets are regulated by nitrate [45].

In this paper, we used Illumina sequencing technology to characterize the poly-A + and sRNA component of nitrate- and control-treated Arabidopsis roots to identify new nitrate-responsive genes. Using bioinformatics analysis of our libraries and miRNA prediction algorithms we were able to find new root expressed genes including new mRNAs and miRNAs. We discovered a new miRNA/target module that might act as an integrator of N and carbon metabolism in Arabidopsis roots.

## Results

### Deep sequence analysis of the root transcriptome

In order to determine poly-A + and sRNA expression of Arabidopsis roots and their changes in response to nitrate, we grew plants in hydroponic nitrate-free medium with 0.5 mM ammonium succinate as the only N-source for two weeks and treated them with 5 mM KNO_3_, or 5 mM KCl as control, for 2 hours. These experimental conditions have been previously shown to elicit robust gene expression responses to nitrate [10,44,45]. Total RNA from two independent sets of plants (biological replicates) was extracted from roots, and poly-A + enriched and sRNA fractions were used to construct libraries for Illumina sequencing (see Methods for details). The sequencing yielded ~5 to 8 million 35 bp long (sRNA libraries) or 50 bp long (poly-A + libraries) raw reads per sample library. After quality control filtering and trimming adaptor sequences (see Methods), the reads were mapped to the *Arabidopsis thaliana* genome using the Arabidopsis genome annotation available at The Arabidopsis Information Resource (TAIR) v.10 (http://www.arabidopsis.org). Approximately two thirds of the total Illumina reads perfectly matched the genome and were used for further analysis (Additional file 1).

**Figure 1:**
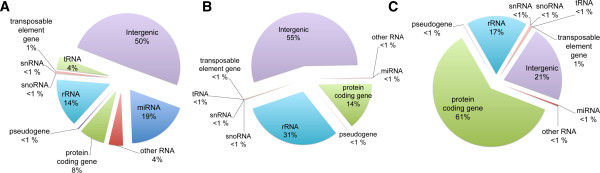
**Categories of genomic origins of sequenced RNAs**. Valid reads were classified according to their annotation in TAIR10 database. We show the percentage of the total valid reads mapping to these regions. The number of reads matching to multiple genomic regions were weighted by the number of loci. **A**. sRNA reads, **B**. Poly-A + reads. **C**. Poly-A + reads that have a single match to the genome.

Analysis of the size distribution of sequences in the sRNA libraries showed that 21 nt long RNA molecules were the most abundant followed by 24 nt long sequences (Additional file 2). The pattern of sRNA sizes reflects a typical population of sRNAs with abundant miRNAs and tasiRNA (21–22 nt) and siRNAs (23–24 nt) (Additional file 2). However, we did not find accumulation of tRNA fragments as described in roots of phosphate-starved plants [38] or nitrate-starved seedlings [33]. We did not observe any obvious effect of nitrate provision on RNA size distribution (Additional file 2), suggesting that nitrate treatments under our experimental conditions do not have a global effect on sRNA population structure. Next, valid sequences were classified according to the genomic regions they match. Most sRNA sequences matched intergenic regions (8,415,076 sequences, 50%), followed by miRNA (3,189,443 sequences, 19%) and rRNA genes (2,469,734 sequences, 14% of the total valid reads) (Figure 1A). We were able to detect 142 distinct mature Arabidopsis miRNA sequences, corresponding to 98 different miRNA families, according to the miRBase database v.17 (http://www.mirbase.org) (Additional file 3). The number of miRNA sequences identified represents 66.7% of the 212 miRNAs reported in miRBase v.17, indicating that a considerable proportion of known miRNAs are expressed in the root organ. This number greatly exceeds the previously reported number of miRNAs expressed in roots, that indicated expressed miRNAs are less than 40% of the annotated total miRNAs [36,46]. We were also able to identify sequences corresponding to trans-acting siRNAs (ta-siRNA), including ta-siRNAs arising from the TAS1, TAS2 and TAS3 genes (Additional file 3). It has recently been shown that a significant number of miRNAs have specific root developmental zone or root cell type expression profiles [47]. Most root miRNAs showed low expression levels under our experimental conditions (Additional file 3), suggesting developmental control or expression in specific cell-types of the Arabidopsis root.

For Illumina libraries made from poly-A + RNA, a considerable amount of sequences map to intergenic regions (9,542,618 sequences, 55% of the reads) (Figure 1B). Inspection of sequences matching intergenic regions showed that most of them arise from telomeric or centromeric regions. Transcription from intergenic zones has been reported in previous high-throughput sequencing and tiling array experiments [48-51]. When we considered sequences with a unique match to the genome, only 732,226 sequences (22%) mapped to intergenic regions (Figure 1C). A high proportion of these sequences is supported by Arabidopsis ESTs or cDNAs (710,814 sequences, 97%) obtained from TAIR.

As shown in Additional file 4, most of these sequences are located near the 5’ or 3’ of annotated genes. We found sequences matching intergenic regions from poly-A + enriched libraries matching the same strand as annotated genes (Additional file 4 A,C). Interestingly, we also found sequences near annotated genes in antisense orientation (Additional file 4 B,D). These could represent novel transcripts that could have a role in controlling the expression of corresponding genes.

Reads matching protein coding genes (2,094,509 sequences) represent ~60% of the unique reads in poly-A + libraries (Figure 1C). The number of expressed protein coding genes detected unambiguously (19,979 protein coding genes) represents 73% of the total annotated in the Arabidopsis genome. Similar to sRNAs, a considerable proportion of genes are expressed in a cell-specific manner [52,53], thus some of the low-expressed transcripts detected under our experimental conditions might be developmentally controlled and/or expressed in specific cell-types of the root.

To date, most transcriptomics studies on the root nitrate response have been performed using the Affymetrix ATH1 GeneChip [8-11,44,45,54]. In order to determine how our sequencing data compares with data obtained with the Affymetrix ATH1 GeneChip, we used the same RNA samples for Illumina library preparation and ATH1 microarray hybridization. We used the *affy* package library from Bioconductor (http://www.bioconductor.org) to determine the number of present calls in the ATH1 microarrays as a measure of gene detection. We were able to find 13,964 probes with a present call, approximately 67% of the gene specific probes that are present in the ATH1 microarray (Additional file 5). The Illumina sequencing data detected 13,411 of these genes (96%, at least one read matching the gene) and 3,022 annotated elements that were called absent in the ATH1 array. We found that these 3,022 elements had low expression values when compared with the 13,411 Illumina-detected elements that had present calls in Affymetrix (Additional file 6A,B). Additionally, Illumina was able to detect 4,215 elements that had no probe on the ATH1 microarray (Additional file 5).

In order to determine how data on nitrate-responsive genes obtained with RNA-seq and Affymetrix ATH1 chips correlated, we calculated the correlation between the KNO_3_/KCl ratio for RMA normalized Affymetrix gene expression and the KNO_3_/KCl ratio obtained for normalized libraries at different average gene coverages (AGCs). We defined AGC as the number of reads matching a gene multiplied by read length and divided by gene length. We found correlation between KNO_3_/KCl ratios increase hyperbolically as average gene coverage increases (Additional file 7). This indicates correlation between the two techniques depends on gene expression levels. We found excellent correlation (r^2^ ≥ 0.9) between RNA-seq and ATH1 arrays when gene coverage was 0.8 or higher (reads matching the gene represent 80% or more of the gene length) (Additional file 7). These results highlight the potential of the sequencing strategy to identify novel nitrate-responsive genes in Arabidopsis roots.

### Deep sequencing reveals a new nitrate-responsive component of the arabidopsis root transcriptome

**Figure 2:**
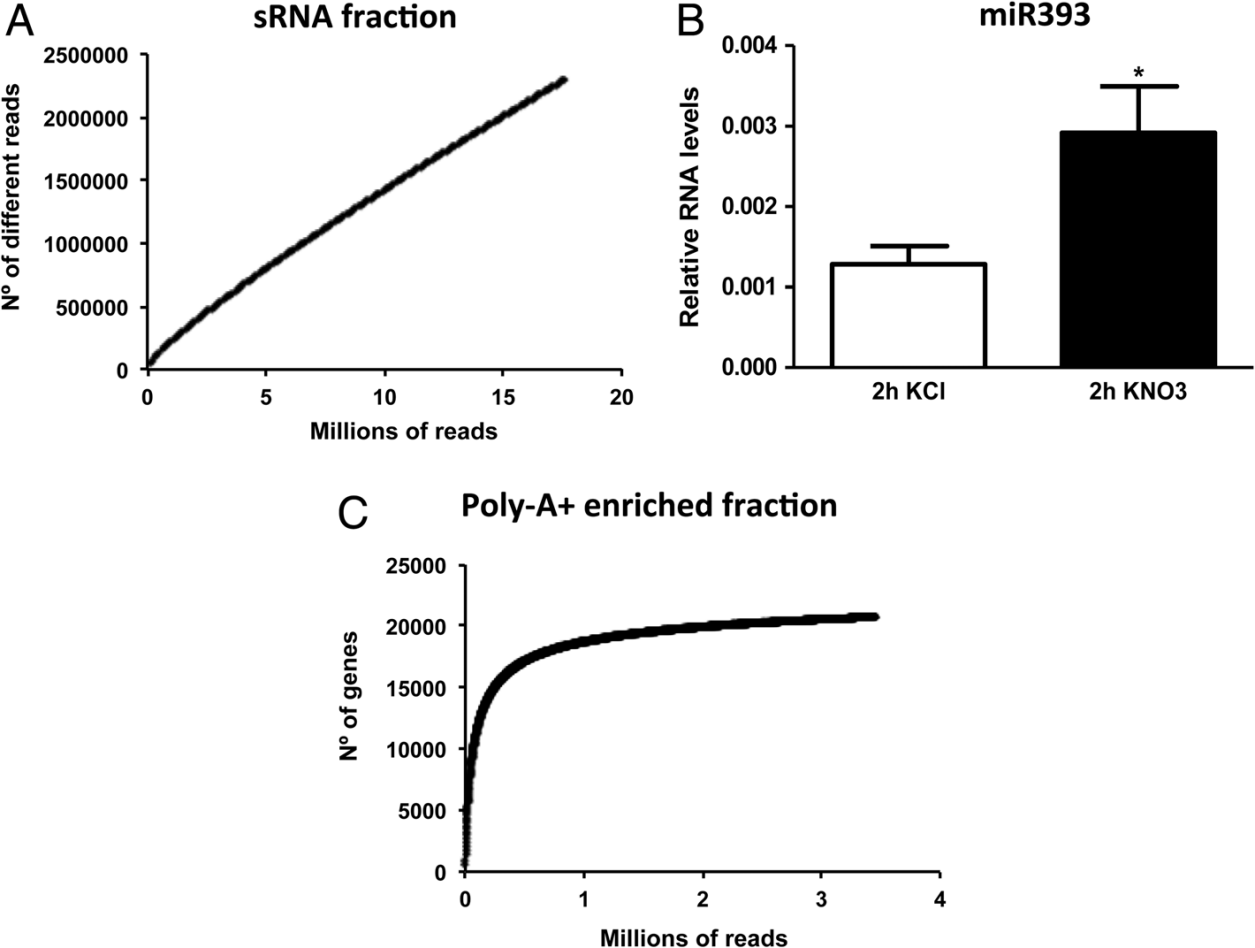
**Analysis of the diversity of sequences suggests that a higher depth of sequencing is required for quantitative results for sRNA libraries**. **A**.Rarefaction curve represents the number of different reads found at the indicated number of reads. The Y axis represents the number of different sequences that could be determined from each sample and the X axis represent the number of random sequences from our sRNA libraries. **B**. miR393 is regulated by nitrate in qRT-PCR experiments. We show results for three biological replicates. We show standard errors for each bar. The asterisk indicates means that differ significantly (p < 0.05). **C**. Rarefaction curve of unique reads that match annotated genes from poly-A + libraries.

In order to identify known miRNAs that are N-regulated under our experimental N-treatment conditions, we used the DESeq package in R to analyze digital gene expression in the RNA-sequencing data [55]. Replicates were used independently for statistical analysis of gene expression. Surprisingly, we were not able to identify known miRNAs (reported in miRBase v17) regulated by nitrate in roots based on our RNA-sequencing data. In order to distinguish between a technical and biological explanation for this result, we calculated an RNA rarefaction curve considering increasing number of random sequences from our sRNA libraries and the number of different sequences that could be determined from each sample (Figure 2A). We found that even when considering the total number of sequences available in our experiments, almost 17 million reads, we were far from saturation. This analysis indicates that most molecules in our sRNA libraries were sequenced only a few times, making it difficult to obtain accurate quantitative results. Using the same RNA samples and quantitative real time PCR, we were able to corroborate induction of miR393 (Figure 2B), a miRNA previously identified as nitrate responsive [37]. This result indicates that a significantly higher depth of sequencing than the current standards [33,34,36,38] is required for quantitative comparison of the sRNA fraction of the Arabidopsis transcriptome. In contrast, when quantifying mRNAs a considerably lower number of sequences is required to reach saturation (Figure 2C).

Among the poly-A + sequences, we found 505 regulated genes, considering only sense sequences that have a unique match to known genes. From these genes, 392 were induced and 113 were repressed by the nitrate treatment (Additional file 8). Regulated genes had an overrepresentation of genes belonging to the “nitrate response”, “nitrate transport”, “nitrate metabolic process”, “nitrate assimilation”, “nitrogen cycle metabolic process” and “cellular nitrogen compound biosynthetic process” Gene Ontology annotation, indicating that RNA-seq was successful for identifying nitrate responsive genes. Among these nitrate-regulated genes, we found 40 protein coding genes without probes in the ATH1 GeneChip (Table 1). We selected eight genes and validated them using real time quantitative reverse transcription polymerase chain reaction (RT-qPCR) (Additional file 9). Among the new nitrate-responsive genes, we found transcription factors and components of signaling cascades such as a γ subunit of the heterotrimeric G protein, AGG2. These genes may represent novel targets in the nitrate regulatory pathways in plants.

**Table 1:** **Illumina sequencing of poly**-**A** + **RNA enriched fraction identifies new nitrate responsive genes**

**AGI identifier**	**Description**	**log**_**2**_ (**KNO**_**3**_/**KCl**)
**AT5G63160**	BT1, BTB and TAZ domain protein 1	5.2
**AT1G11655**	Unknown protein	4.3
**AT5G65030**	Unknown protein	4.2
**AT1G70260**	nodulin MtN21 /EamA-like transporter family protein	4.0
**AT2G33550**	Homeodomain-like superfamily protein	3.7
**AT1G68238**	Unknown protein	3.7
**AT4G34419**	Unknown protein	3.6
**AT1G02030**	C2H2-like zinc finger protein	3.1
**AT4G34800**	SAUR-like auxin-responsive protein family	3.0
**AT5G03330**	Cysteine proteinases superfamily protein	2.9
**AT1G60050**	Nodulin MtN21 /EamA-like transporter family protein	2.9
**AT2G45760**	BAL, BAP2, BON association protein 2	2.8
**AT1G70800**	Calcium-dependent lipid-binding (CaLB domain) family protein	2.8
**AT4G29905**	Unknown protein	2.7
**AT3G22942**	AGG2, G-protein gamma subunit 2	2.2
**AT1G23149**	CPuORF29, conserved peptide upstream open reading frame 29	2.2
**AT1G23150**	Unknown protein	2.2
**AT5G65980**	Auxin efflux carrier family protein	2.2
**AT3G14260**	Protein of Unknown function (DUF567)	2.1
**AT3G48180**	Unknown protein	2.1
**AT3G25717**	DVL6, RTFL16, ROTUNDIFOLIA like 16	2.1
**AT2G41440**	Unknown protein	2.1
**AT1G13245**	DVL4, RTFL17, ROTUNDIFOLIA like 17	2.0
**AT1G68825**	DVL5, RTFL15, ROTUNDIFOLIA like 15	2.0
**AT5G58320**	Kinase interacting (KIP1-like) family protein	2.0
**AT3G29034**	Unknown protein	1.9
**AT1G22882**	Galactose-binding protein	1.6
**AT4G04745**	Unknown protein	1.6
**AT4G09180**	basic helix-loop-helix (bHLH) DNA-binding superfamily protein	1.3
**AT1G45249**	ABF2, abscisic acid responsive elements-binding factor 2	1.3
**AT5G38200**	Class I glutamine amidotransferase-like superfamily protein	1.2
**AT2G18193**	P-loop containing nucleoside triphosphate hydrolases superfamily protein	1.2
**AT5G10200**	ARM-repeat/Tetratricopeptide repeat (TPR)-like protein	1.2
**AT5G52882**	P-loop containing nucleoside triphosphate hydrolases superfamily protein	0.9
**AT2G31141**	Unknown protein	0.8
**AT3G48340**	Cysteine proteinases superfamily protein	−1.0
**AT2G23790**	Protein of Unknown function (DUF607)	−1.2
**AT1G52120**	Mannose-binding lectin superfamily protein	−1.5
**AT4G39795**	Protein of Unknown function (DUF581)	−1.9
**AT3G06550**	O-acetyltransferase family protein	−2.0

Reads were mapped to the Arabidopsis genome and regulated genes were determined using DESeq. We identified protein coding genes that were not represented on the Affymetrix ATH1 microarray. We show the log_2_(KNO_3_/KCl) value for 2 biological replicates.

### Prediction of new genes

**Figure 3:**
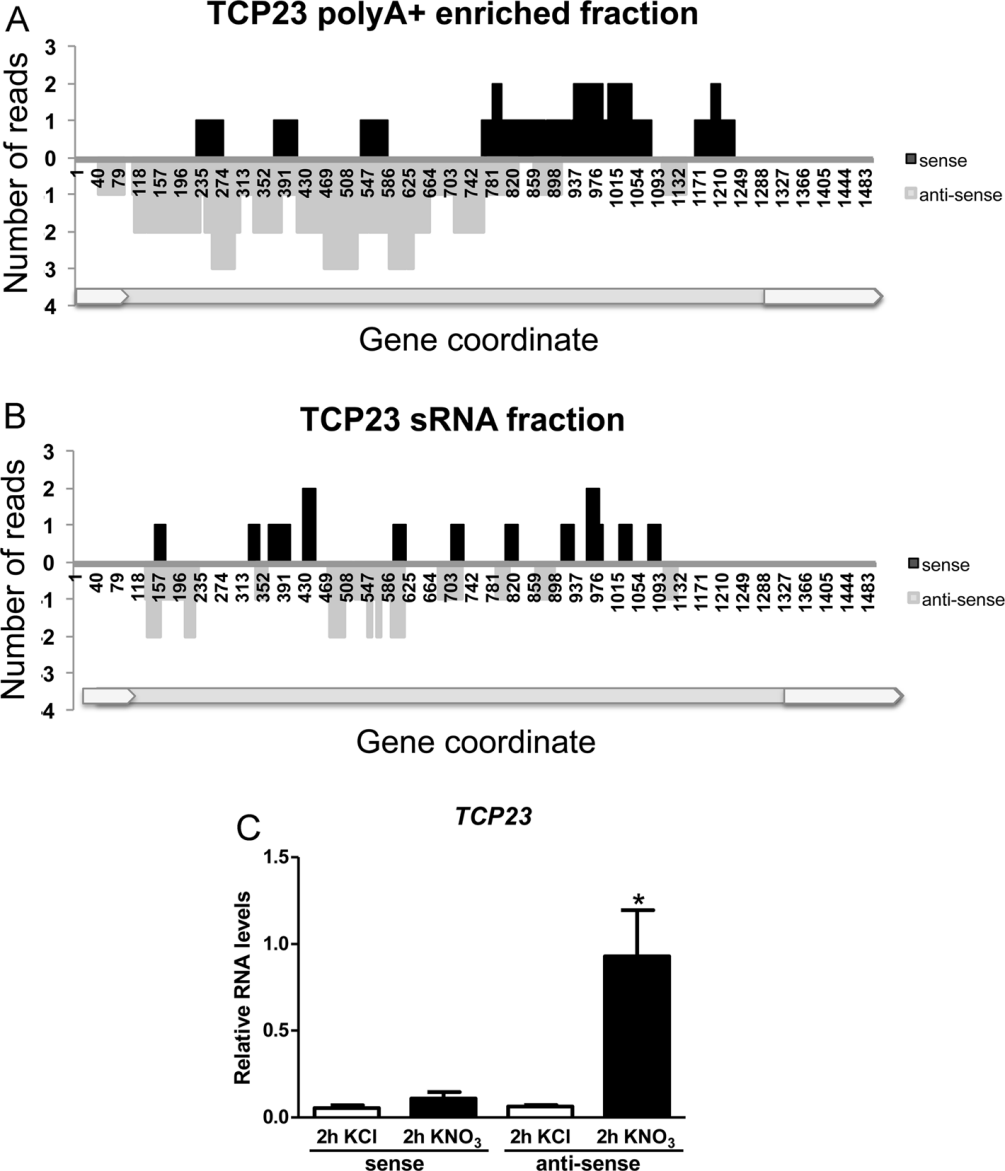
**TCP23as is a novel nitrate**-**regulated gene that is anti**-**sense to TCP23**. **A**. We represent RNA poly-A + transcripts mapping the TCP23 (AT1G35560) region. The black and grey bars represent reads sense or antisense to AT1G35560 respectively. We show the gene structure of TCP23 in gray. Gray represents 5’UTR and 3’UTR and dark gray represents the coding region. **B**. We represent sRNA transcripts mapping the TCP23 locus similar to panel A. **C**. cDNA was prepared using strand-specific primers for TCP23 and TCP23as. We quantified relative RNA levels of both transcripts using RT-qPCR. We show the results of three biological replicates and standard error.

In order to identify regions of the Arabidopsis genome that could encode new genes expressed under our experimental nitrate-treatment conditions, we searched for clusters of sequences that match the genome uniquely in regions without annotation (see Methods). These clusters could overlap annotated genes but in anti-sense orientation. Average exon length in the Arabidopsis genome (TAIRv10) is 298 nt, therefore we only considered clusters of 300 nt or more. We found 17 clusters with these criteria (Additional file 10), 4 of which were located in the complementary strand of annotated genes and might represent natural antisense transcripts (NATs). Two clusters have been previously reported as cis-NATs in an analysis of Arabidopsis full-length cDNAs, At5g49440 and At3g19380 [56]. We found that one of the 17 clusters was induced by nitrate treatments. We labeled this cluster TCP23as as it is antisense to the TCP transcription factor TCP23 (At1g35560) (Figure 3A). We also found sRNAs matching the same region both in sense and antisense orientation (Figure 3B), however we did not find a correlation between their expression and TCP23as regulation by nitrate, suggesting these sequences most likely represent degradation products of TCP23 and TCP23as. TCP23 was found not to be regulated by nitrate in our RNA-sequencing data (Additional file 5). In order to validate expression of this putative antisense transcript, we reverse-transcribed root RNA using strand-specific primers for TCP23 and for its antisense transcript and performed PCR with gene specific primers. As shown in Figure 3C, both TCP23 and TCP23as are expressed in roots, but only TCP23as is induced by the nitrate treatment. Our data suggest TCP23as could represent a novel nitrate-regulated transcript that might regulate TCP23 expression at the transcriptional or post-transcriptional level. Given the low expression levels of TCP23 in whole root sample, it is likely that its regulation by TCP23as occurs only in a subset of root cells.

### Prediction of novel miRNA genes

Numerous approaches have been utilized to predict and discover miRNAs [57,58]. However, few experiments have been performed under contrasting N nutrient conditions [33,34,36]. To generate a list of putative new miRNAs that may be expressed under our experimental conditions, we used the miRNA gene prediction tool available in the University of East Anglia (UEA) sRNA toolkit, miRCat (http://srna-tools.cmp.uea.ac.uk) [59]. We chose this prediction tool because it is optimized for the identification of plant miRNA hairpins, and it has been trained and tested with published *Arabidopsis thaliana* high-throughput sRNA sequence data. We used as input for miRCat the filtered sRNA sequences obtained from our 4 sRNA libraries. miRCat was able to predict 123 mature miRNA sequences corresponding to 87% of the known miRNAs identified in our samples, indicating that the prediction algorithms implemented in miRCat are highly efficient in identifying plant miRNAs. The miRCat program was able to predict 51 new miRNA sequences when compared with miRBase v17 (Table 2). From these 51 new miRNA sequences our studies uncovered, 12 were recently cross-validated by other groups, suggesting the veracity of our results [47,60-63]. 21 putative new miRNA sequences were found in intergenic regions, likely representing new transcriptional units (Table 2). 10 miRNA sequences were found inside introns and 2 were found in the 5’UTR of protein coding genes suggesting they are transcribed along with the gene they overlap and 1 miRNA was found in a pseudogene (Table 2). 17 new miRNA sequences were located inside the region coding for the stem loop of known miRNAs (Table 2). Sequences that map onto miRNA precursors and that do not correspond to the mature miRNA or miRNA* sequences have been previously reported in Arabidopsis and are potentially functional miRNAs that are generated by the miRNA pathway [64,65]. All these new miRNAs have low expression levels, most of them being sequenced less than 50 times in our libraries, which probably explains why they have not been reported previously.

**Table 2:** Illumina sequencing identifies novel miRNAs

**miRNA**	**Chr**	**miRNA Start**	**miRNA End**	**Mature Sequence**	miRNA*	**miRNA previoulsy reported**	**miRNA located in**
miR5640	1(−)	1653540	1653560	AUGAGAGAAGGAAUUAGAUUC	YES	ath-miR5640 [47]	AT1G05570.1 intron
ath-MIR472-5p	1(−)	4182266	4182286	AUGGUCGAAGUAGGCAAAAUC	NO	Novel	ath-MIR472 stem loop
ath-MIR8166	1(−)	4525316	4525337	AGAGAGUGUAGAAAGUUUCUCA	NO	Novel	Intergenic region AT1G13240-AT1G13245
miR5654-3p	1(+)	11786350	11786371	GAAGAUGCUUUGGGAUUUAUUU	NO	miR5654-3p [47,63]	AT1G32583.1, 5'UTR
ath-MIR829-5p	1(−)	11834153	11834173	ACUUUGAAGCUUUGAUUUGAA	YES	Novel	ath-MIR829 stem loop
miR5014a	1(+)	24554009	24554029	UGUUGUACAAAUUUAAGUGUA	YES	ath-miR5014a [47,60]	AT1G65960.1 intron
ath-MIR840-3p	1(−)	771385	771405	UUGUUUAGGUCCCUUAGUUUC	YES	Novel	ath-MIR840 stem loop
ath-MIR398a-3p	2(+)	1040948	1040968	AAGGAGUGGCAUGUGAACACA	YES	Novel	ath-MIR398a stem loop
ath-MIR8180	2(+)	2063980	2063998	UGCGGUGCGGGAGAAGUGC	NO	Novel	Intergenic region AT2G05580-AT2G05590
ath-MIR8175	2(+)	3740938	3740957	GAUCCCCGGCAACGGCGCCA	NO	Novel	Intergenic region AT2G09880-AT2G09890
ath-MIR396a-3p	2(−)	4142331	4142351	GUUCAAUAAAGCUGUGGGAAG	YES	Novel	ath-MIR396a stem loop
ath-MIR8168	2(+)	5080690	5080710	AGGUGCUGAGUGUGCUAGUGC	NO	Novel	Intergenic region AT2G12490-AT2G12500
ath-MIR5632-5p	2(−)	8392588	8392608	UUGAUUCUCUUAUCCAACUGU	YES	Novel	ath-MIR5632 stem loop
ath-MIR8167a	2(+)	8894985	8895006	AGAUGUGGAGAUCGUGGGGAUG	NO	Novel	Intergenic region AT2G20620-AT2G20625
miR5995b	2(−)	10026977	10026997	AAAGAUGCAGAUCAUAUGUCC	YES	ath-miR5995b [63])	Intergenic region AT2G23540-AT2G23550
ath-MIR831-5p	2(+)	10247259	10247280	AGAAGCGUACAAGGAGAUGAGG	NO	Novel	ath-MIR831 stem loop
miR5637	2(−)	12270195	12270216	UAGAGGAAAAUAUAGAGUUGGG	NO	ath-miR5637 [47]	Intergenic region AT2G28620-AT2G28625
ath-MIR8170.1	2(+)	14100020	14100040	AUAGCAAAUCGAUAAGCAAUG	YES	Novel	AT2G33255.1 intron
ath-MIR8170.2	2(+)	14100079	14100099	UUGCUUAAAGAUUUUCUAUGU	YES	Novel	AT2G33255.1 intron
ath-MIR160a-3p	2(+)	16340342	16340362	GCGUAUGAGGAGCCAUGCAUA	YES	Novel	ath-MIR160a stem loop
ath-MIR8171	2(+)	16890466	16890486	AUAGGUGGGCCAGUGGUAGGA	NO	Novel	AT2G40440.1 intron
ath-MIR166a-5p	2(+)	19176128	19176148	GGACUGUUGUCUGGCUCGAGG	YES	Novel	ath-MIR166 stem loop
ath-MIR408-5p	2(+)	19319866	19319886	ACAGGGAACAAGCAGAGCAUG	YES	Novel	ath-MIR408 stem loop
miR5650	2(+)	19686959	19686979	UUGUUUUGGAUCUUAGAUACA	YES	ath-miR5650 [47]	AT2G48140.1 intron
miR173-5p	3(+)	8236161	8236182	UUCGCUUGCAGAGAGAAAUCAC	YES	ath-miR173-5p [62,63]	ath-miR173-5p stem loop
ath-MIR8169	3(+)	8836359	8836379	AUAGACAGAGUCACUCACAGA	NO	Novel	Intergenic region AT3G24340-AT3G24350
ath-MIR8183	3(−)	11747799	11747819	UUUAGUUGACGGAAUUGUGGC	NO	Novel	AT3G30110.1, pseudogene
ath-MIR8165	3(−)	16538510	16538530	AAUGGAGGCAAGUGUGAAGGA	NO	Novel	Intergenic region AT3G45170-AT3G45180
ath-MIR8174	3(−)	16589431	16589451	AUGUGUAUAGGGAAGCUAAUC	NO	Novel	Intergenic region AT5G38460-AT5G38470
miR5651	3(+)	17178489	17178509	UUGUGCGGUUCAAAUAGUAAC	YES	ath-miR5651 [47]	Intergenic region AT3G46616-AT3G46620
ath-MIR8167b	3(−)	8894985	8895006	AGAUGUGGAGAUCGUGGGGAUG	NO	Novel	Intergenic region AT3G47410-AT3G47420
ath-MIR8167c	3(−)	17469946	17469967	AGAUGUGGAGAUCGUGGGGAUG	NO	Novel	Intergenic region AT3G50700-AT3G50710
miR5633	3(+)	19544786	19544807	AUGAUCAUCAGAAAACAGUGAU	NO	ath-miR5633 [47])	Intergenic region AT3G52730-AT3G52740
ath-MIR393b-3p	3(+)	20691778	20691798	AUCAUGCGAUCUCUUUGGAUU	YES	Novel	ath-MIR393 stem loop
ath-MIR8182	3(+)	22678166	22678187	UUGUGUUGCGUUUCUGUUGAUU	NO	Novel	AT3G61270.1, 5'UTR
ath-MIR166b-5p	3(+)	22922212	22922232	GGACUGUUGUCUGGCUCGAGG	YES	Novel	ath-MIR166 stem loop
ath-MIR8172	4(−)	7102572	7102592	AUGGAUCAUCUAGAUGGAGAU	YES	Novel	Intergenic region AT4G11800-AT4G11810
ath-MIR8179	4(−)	7161930	7161950	UGACUGCAUUAACUUGAUCGU	NO	Novel	AT4G1192.1 intron
ath-MIR8176	4(+)	11795199	11795219	GGCCGGUGGUCGCGAGAGGGA	NO	Novel	Intergenic region AT4G22320-AT4G22330
ath-MIR8178	4(+)	18087285	18087305	UAACAGAGUAAUUGUACAGUG	NO	Novel	AT4G38760.1 intron
ath-MIR8184	5(−)	3311974	3311994	UUUGGUCUGAUUACGAAUGUA	NO	Novel	Intergenic region AT5G10504-AT5G10510
miR5629	5(+)	3802933	3802954	UUAGGGUAGUUAACGGAAGUUA	NO	ath-miR5629 [47]	Intergenic region AT5G11790-AT5G11800
ath-MIR865.2	5(+)	5169992	5170011	UCUGGGAUGAAUUUGGAUCU	NO	Novel	ath-MIR865 stem loop
miR1888	5(+)	7168879	7168899	UAAGUUAAGAUUUGUGAAGAA	NO	ath-miR1888 [61,62]	AT5G21100.1 intron
ath-MIR8173	5(−)	7478572	7478592	AUGUGCUGAUUCGAGGUGGGA	NO	Novel	Intergenic region AT5G22510-AT5G22520
ath-MIR8177	5(−)	9362634	9362655	GUGUGAUGAUGUGUCAUUUAUA	NO	Novel	Intergenic region AT5G26617-AT5G26620
miR5638b	5(+)	14100017	14100037	ACAGUGGUCAUCUGGUGGGCU	NO	ath-miR5638b [47]	Intergenic region AT5G35945-AT5G35950
ath-MIR160c-3p	5(−)	19009095	19009115	CGUACAAGGAGUCAAGCAUGA	YES	Novel	ath-MIR160c stem loop
ath-MIR870-5p.1	5(−)	21395592	21395612	UUAGAAUGUGAUGCAAAACUU	NO	Novel	ath-MIR870 stem loop
ath-MIR870-5p.2	5(−)	21395604	21395624	AAGAACAUCAAAUUAGAAUGU	NO	Novel	ath-MIR870 stem loop
ath-MIR8181	5(−)	21641289	21641308	UGGGGGUGGGGGGGUGACAG	NO	Novel	AT5G5333.1, intron

Sequences from the Illumina libraries were queried for sequences representing putative new miRNAs with the miRCat program from the UEA sRNA toolkit. We show the genomic location and sequences of these miRNAs. Presence of reads corresponding to miRNA* are indicated (Yes or No). miRNAs that are not included in miRBase 17 but that have been cross-validated by other groups are indicated.

The name of mature sequences derived from the same arm of known hairpin precursor were named with the suffix .1 or .2. If the sequences derive from the opposite arm from the previously annotated sequence,were named with the suffix -5p, -3p.

### A novel nitrate-responsive miRNA/target regulatory module (AtPPC3/miR5640)

**Figure 4:**
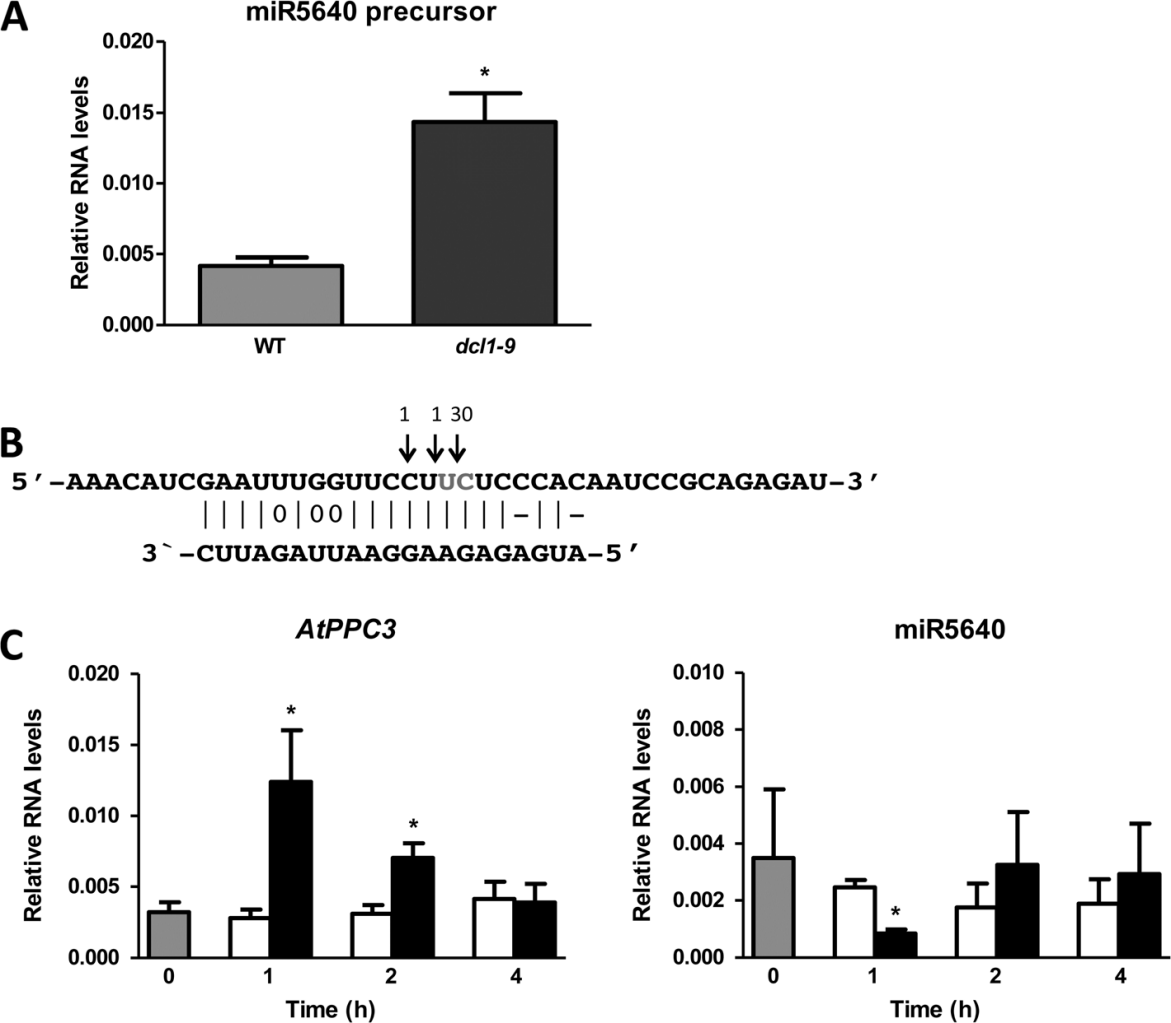
**Illumina sequencing identifies a novel miRNA**/**TARGET module consisting of miR5640 and its target**, ***AtPPC3***. **A**. We analyzed the RNA levels of miR5640 predicted precursor using RT-qPCR in WT plants and in the *dcl1*-*9* mutant. **B**. We used RLM-RACE to validate *AtPPC3* as target of miR5640. The arrows show the numbers of colonies found with the cleavage product. **C**. We determined the RNA levels of *AtPPC3* and of mature miR5640 after 1, 2 and 4 hours of nitrate (black bars) or KCl (white bars) treatments. We show the results of three biological replicates and standard error.

In order to further characterize the role of the novel miRNAs in the root nitrate response, we predicted target genes for new miRNAs sequenced in our libraries using the target prediction tool Target finder from the UEA sRNA toolkit, (Additional file 11). The program is based on a set of rules determined specifically for plant miRNA/TARGET interactions [66,67]. We looked in the target list for genes that could be related to N metabolism or to root growth regulation and that were either induced or repressed by nitrate based on our Illumina results. One of the predicted targets was the transcript for PHOSPHOENOL PYRUVATE CARBOXYLASE 3 (AtPPC3, At3g14940) (Additional file 11), an enzyme that catalyzes CO_2_ incorporation with phosphoenol pyruvate to form oxaloacetate [68]. *AtPPC3* is induced in roots after nitrate treatment based on our sequencing data (Additional file 8). The miRNA predicted to target *AtPPC3* has recently been reported as miR5640 [47]. miR5640 has been shown to be expressed in Arabidopsis primary root in the apical half of the meristematic zone (early meristematic zone), the elongation zone, and the maturation zone, according to sequencing data, but no additional validation on its expression or additional characterization of its function or target prediction has been performed [47]. In order to validate miR5640 as a *bona fide* miRNA, we confirmed its expression and expression of its precursor in roots using RT-qPCR. In addition, miR5640 precursor accumulated in the DCL1 (*dcl1*-*9*) mutant plants (Figure 4A), indicating that miR5640 precursor is processed by DCL1 as most miRNA precursors [69]. In order to experimentally confirm that AtPPC3 is a miR5640 target and to map the miR5640 cleavage site, we performed a modified RLM-RACE procedure [70]. We were able to detect and clone an amplification product corresponding to the expected size of a miR5640-cleaved AtPPC3 fragment. It has been described that cleavage of the target transcripts occurs near the middle of the base-pairing interaction [71,72]. As shown in Figure 4B, 30 out of 32 clones sequenced had a cleavage site inside the miRNA complementary sequence, between the 8^th^ and 9^th^ complementary bases from the miRNA 5’ end. This result suggests that *AtPPC3* is a target of miR5640 and further corroborates miR5640 as a *bona fide* miRNA. Based on our sequencing data, we did not find differential expression of miR5640 2 hours after nitrate treatment, although *AtPPC3* is induced by this treatment. In order to determine if miR5640/AtPPC3 could represent a nitrate-responsive miRNA/TARGET module, we analyzed the nitrate response of the miR5640/AtPPC3 pair on a time course using RT-qPCR. As shown in Figure 4C, *AtPPC3* peak of induction by nitrate correlates with miR5640 repression by nitrate. The reduction of *AtPPC3* levels over time also correlates with the de-repression of miR5640, suggesting that *AtPPC3* levels are post-transcriptionally regulated by this miRNA in response to nitrate. Thus, miR5640/AtPPC3 represents a nitrate-responsive module that could be important for modulating carbon/N balance for nitrate assimilation in Arabidopsis roots.

## Discussion

High throughput sequencing approaches have become powerful tools to identify the transcriptome of Arabidopsis and other systems. Besides the ability to profile novel genes expressed at low levels which could not be identified by traditional cloning and sequencing approaches, the high depth of sequencing obtained by these techniques allows for the absolute quantification of genes, and the comparison of gene expression under different experimental conditions [38,73,74]. Our high throughput sequencing results provided a detailed view of poly-A + RNAs and sRNAs expressed in Arabidopsis roots. We found that roots express a considerable portion of known protein coding genes and miRNA genes. However, most of these genes are expressed at low levels. These transcripts might represent cell specific transcripts whose expression is diluted when considering the whole root. Transcriptomics analysis of specific root cell types has shown that gene expression has an important cell-specific component that gives rise to functional diversification of cells [52,53].

Even though the sequencing depth used to characterize the sRNA component did not allow for accurate quantitative estimates, we were able to discover novel miRNAs that have eluded previous efforts. Our bioinformatics analysis predicted 51 putative miRNAs expressed in roots under the experimental conditions. Most of these sequences were poorly expressed with less than 1 transcript per million transcripts. A recent publication that analyzes miRNA expressed in specific developmental zones and cell types of the root shows that 9 of these new miRNAs have cell or developmental zone specific expression [47] which can explain their low expression in the whole root samples. We were able to validate one of the predicted miRNAs, miR5640, as a putative miRNA expressed in roots. This miRNA is located inside intron 23 of the *CALLOSE SYNTHASE 1* gene (*CALS1*, AT1G05570). Intronic miRNAs represent the majority of the miRNAs of animal systems but there are only a few examples in Arabidopsis [75,76]. Characterized intronic Arabidopsis miRNAs include miR162a and miR838 which are involved in the regulation of *DCL1*[24,77,78]. However, analyzing our sequencing results, we found that the CALS1 transcript was not regulated by nitrate, thus miR5640 could have an independent nitrate-responsive promoter or pri-miR5640 processing to generate the mature miRNA could be a nitrate-regulated process.

We found miR5640 targeted the transcript that codes for *AtPPC3*, one of the four phosphoenolpyruvate carboxylase enzymes in Arabidopsis [79]. AtPPCs are important enzymes of carbon metabolism that catalyze the β-carboxylation of phosphoenolpyruvate to yield oxaloacetate. In C3 plants and algae, it has been shown that ATPPCs are important for the production of carbon skeletons for nitrogen assimilation [68,80,81]. Although there has been an extensive biochemical characterization of the AtPPCs enzymes in Arabidopsis, there are no reports of their function in N metabolism. AtPPC3 is a root specific AtPPC [82] and we found that it was nitrate-induced in our experiments, which is in agreement with the positive effect on nitrate assimilation predicted for this AtPPC. We also found evidence indicating that nitrate induction of *AtPPC3* might depend on a miR5640-mediated post-transcriptional regulation of *AtPPC3* levels in response to nitrate. Although we found AtPPC3 cleavage products that might be generated by miR5640 action over this transcript, we need further experiments to validate *AtPPC3* as a miR5640 target (i.e. to analyze *AtPPC3* levels in a miR5640 overexpressor plant), and to validate the role of this miRNA/*TARGET* module in nitrate assimilation in roots.

An advantage of using high throughput sequencing is the ability to interrogate gene expression without the representation bias present in microarray experiments. We discovered 40 protein-coding genes that have not been reported to be nitrate-responsive in previous transcriptomics analysis of Arabidopsis roots. Among them, we found highly responsive genes such as *BT1* (At5g63160), a calmodulin-binding scaffold protein that acts redundantly with other BT proteins in female gametophyte development [83]. The closest homolog of BT1, BT2, has been reported to be responsive to multiple hormonal, stress and nutritional signals, including nitrate [84]. Interestingly, *BT1* is only expressed when nitrate is supplied, suggesting that it might have a nitrate-specific function in roots. The *AGG2* gene, one of the two genes encoding the gamma subunit of heterotrimeric G protein was also induced by nitrate. Heterotrimeric G protein in Arabidopsis has been involved in various developmental processes. In roots, it is involved in lateral root formation [85] and root apical meristem growth [86]. We have found that nitrate has an effect in primary and lateral root growth [37], thus nitrate regulation of *AGG2* might contribute to this response.

NATs are transcripts that fully or partially overlap with other transcripts. These pairs can mediate production of siRNAs to silence gene expression [87]. Additionally, NATs can modulate transcription, can affect mRNA stability and translation and can induce chromatin and DNA epigenetic changes [88]. Computational predictions have shown that the Arabidopsis genome potentially encodes sense-antisense transcript pairs representing approximately 7% of the protein coding genes [56]. We were able to identify 4 putative NATs of >300 bp in our sequencing data. One of these NATs was antisense to *TCP23* gene and was induced by nitrate. *TCP* genes are transcription factors that promote growth and proliferation [89]. TCP23 is predicted to contain a chloroplast-targeting peptide, suggesting it might control transcription of chloroplast genes [90]. Although TCP23 has no described function, other class I TCP factors have been shown to be expressed in meristematic tissues and to control cell cycle genes such as *PCNA* and *CYCB1*;*1*[91,92]. Thus, *TCP23as* induction by nitrate might repress *TCP23* expression, controlling meristematic activity of the primary root. However, further studies are needed to analyze *TCP23as* role over *TCP23* expression on roots and on *TCP23* regulation by nitrate.

## Conclusions

In summary, the sequencing of small RNAs and mRNAs uncovered new genes, and enabled us to develop new hypotheses for nitrate regulation and coordination of carbon and N metabolism. A highlight is the discovery of a novel microRNA, miR5640 and its target, *AtPPC3*. The data suggest that the nitrate-responsive miRNA/target module might be involved in controlling carbon flux to assimilate nitrate into amino acids. These findings suggest that microRNAs can have metabolic regulatory functions, as well as previously described developmental functions [37,44] in the nitrate response of Arabidopsis roots.

## Methods

### Growth and treatment conditions

Approximately 1,500 Arabidopsis seedlings were grown hydroponically on Phytatrays on MS-modified basal salt media without N (Phytotechnology Laboratories, M531) supplemented with 0.5 mM ammonium succinate and 3 mM sucrose under a photoperiod of 16 h of light and 8 h of darkness and a temperature of 22°C using a plant growth incubator (Percival Scientific, Inc.). After 2 weeks, plants were treated with 5 mM KNO_3_ or 5 mM KCl as control for 2 hours.

### Preparation of illumina libraries

Total RNA from from nitrate-treated or control roots was extracted using Trizol® (Invitrogen, cat. Number 15596–026). For poly-A + libraries, poly-A + RNA was enriched using the Poly(A)Purist™ MAG Kit (Ambion, cat, number AM1922M). Poly-A + RNA was decapped using tobacco acid pyrophosphatase and fragmented using RNA Fragmentation Reagents (Ambion, cat. Number AM8740). Low molecular weight RNA (<40 nt) was isolated from 100 μg of total RNA by PAGE on a FlashPAGE™ fractionator (Ambion, cat. Number AM13100). For construction of the libraries, cloning linker (AMP-5’p = 5’pCTG TAG GCA CCA TCA ATdideoxyC-3’) was ligated to the 3’ end of the RNA followed by purification of the ligation product on a 15% polyacrilamide/urea gel. The 3’-ligated product was ligated to the 5’ Solexa linker (5’-rArCrA rCrUrC rUrUrU rCrCrC rUrArC rArCrG rArCrG rCrUrC rUrUrC rCrGrA rUrC-3’). RNA with ligated adaptors was reverse transcribed into DNA using Illumina specific primer (5’- CAA GCA GAA GAC GGC ATA CGA TTG ATG GTG CCT ACA G-3’) and cDNA was then PCR amplified using this primer and a specific primer (5’- AAT GAT ACG GCG ACC ACC GAA CAC TGT TTC CCT ACA CGA CG-3’). The libraries were gel purified using the QIAquick gel extraction kit (QIAGEN, cat. Number 28704). Libraries were sequenced on the Illumina 1G Genome analyzer.

### Sequence analysis

Raw sequences from the Illumina 1G Genome analyzer in FASTQ format were analyzed with publicly available tools. Low quality reads were extracted with fastq quality filter by FASTX toolkit version 0.0.13 (http://hannonlab.cshl.edu/fastx_toolkit/). The Phred quality score was set to 20, a probability of incorrect base call of 1 in 100. 3’ adaptor sequences were trimmed from the Illumina reads, and then were mapped to the Arabidopsis TAIR10 genome using Novoalign version 2.05.17 (http://www.novocraft.com). Perfect match sequences having passed the quality control, polynucleotide filter, and size filter (between 18 and 28 nt for sRNA libraries and ≥18 nt for poly-A + libraries) were selected for further analysis with custom made PERL scripts.

### Determination of differentially expressed genes

To evaluate differential gene expression between KNO_3_ and KCl treated samples, we used sequence counts corresponding to sRNAs or annotated elements as input for the DESeq package version 1.1.6 [55] available from Bioconductor (http://www.bioconductor.org). This tool uses a negative binomial distribution model to test for differential gene expression [55]. We found correlation values of 0.91 and 0.96 for controls and treatments respectively for sRNA-seq and of 0.99 for controls and treatments for RNA-seq data. Replicates were used independently for statistical analysis of gene expression. We adjusted for multiple testing using FDR correction [93] and filtered genes whose expression changed with corrected p-values ≥ 0.05.

### New miRNA and target predictions

Quality filtered Illumina sequences were used as input for the MIRCAT tool [59], available at the University of East Anglia (UEA) sRNA toolkit (http://srna-tools.cmp.uea.ac.uk) using default parameters. To predict miRNA targets, we used the target prediction tool available from the UEA sRNA toolkit. The predicted targets, along with the putative cleavage site on these targets, were further validated using RNAhybrid version 2.1 [94].

### Predicting novel transcribed regions

Novoalign alignments that did not overlap with annotated regions of the genome were pooled from all samples. Regions with continuous alignments in the same strand greater than 300 bp were identified as candidate novel transcribed regions.

### Gene expression analysis using RT-qPCR

Gene expression analysis was carried out using the Brilliant® SYBR® Green QPCR Reagents on a Stratagene MX3000P qPCR system (Agilent) according to manufacturer’s instructions. The RNA levels were normalized relative to the Clathrin adaptor complexes medium subunit family protein (At4g24550). Quantification of microRNA levels was carried out using the High-Specificity miRNA QRT-PCR Detection Kit from Stratagene on a Stratagene MX3000P qPCR system. The RNA levels were normalized relative to U6 snRNA (At3g14735). A list of RT-qPCR primers used in this work is provided in Additional file 12.

### RLM-RACE

A modified procedure for RLM-RACE [70] was carried out using the GeneRacer™ kit. The GeneRacer RNA Oligo adapter was directly ligated to 250 ng of Poly-A + mRNA and the GeneRacer OligodT primer was used to synthesize first strand cDNA. This cDNA was subjected to a PCR amplification procedure with the GeneRacer 5′Primer and the GeneRacer 3′Primer to generate a pool of non-genespecific RACE products. Gene-specific 5′RACE reactions were performed with the GeneRacer 5′Nested Primer and a reverse gene-specific primer. The expected size of the PCR amplicons was checked on a 3% agarose gel. PCR products were cloned and sequenced to confirm predicted miRNA-mediated cleavage of the transcripts.

## Availability of supporting data

The data sets supporting the results of this article are available in the NCBI GEO database [95] repository, under accession GSE44062.

## Competing interests

The authors declare that they have no competing interests.

## Authors’ contributions

EAV participated in the design of the study, performed molecular biology experiments and statistical analyses and wrote the paper. TCM performed the computational analysis of the data, performed the experiments and wrote the paper. GK participated in the design of the study, performed experiments, analyzed the data and helped to draft the manuscript. MK performed computational analysis of the data and helped to draft the manuscript. MT generated the libraries for sequencing and helped to draft the manuscript. WRM participated in sequencing of the libraries. GMC participated in the design and coordination of the study and helped to draft the manuscript. RAG participated in the design and coordination of the study and wrote the paper. All authors read and approved the final manuscript.

## Supplementary Material

Additional file 1 Statistics of filtered sRNA and mRNA reads. Click here for file

Additional file 2 **Global profiling of Illumina**-**sequenced sRNA.** Click here for file

Additional file 3 **Known miRNA and ta**-**siRNA expressed in roots.** Click here for file

Additional file 4 **mRNA reads that unambigoulsly match intergenic regions are located near 5**’ **and 3**’ **of annotated genes.** Click here for file

Additional file 5 **Expression analysis of annotated elements of TAIR v.10.** Click here for file

Additional file 6 **Gene expression distribution of genes represented in the ATH1 microarray in Illumina poly-A + libraries.** Click here for file

Additional file 7 **Correlation between nitrate**-**regulated genes obtained by RNA**-**seq and Affymetrix ATH1 microarrays depends on Average gene coverage.** Click here for file

Additional file 8 Nitrate regulated genes determined using Illumina deep sequencing. Click here for file

Additional file 9 **Deep sequencing of the Arabidopsis root poly-A + enriched fraction identifies new nitrate-regulated genes.** Click here for file

Additional file 10 Identification of putative new genes. Click here for file

Additional file 11 Prediction of putative targets for novel miRNA sequences. Click here for file

Additional file 12 **List of primers used for qRT-PCR.** Click here for file
