# Supplementary material for: Integrated RNA-seq and sRNA-seq analysis identifies novel nitrate-responsive genes in Arabidopsis thaliana roots
Source: BMC Genomics. 2013 Oct 11;14:701. doi: 10.1186/1471-2164-14-701 (PMC3906980; doi:10.1186/1471-2164-14-701)
Supplement: Additional file 1 — Statistics of filtered sRNA and mRNA reads. [file 1471-2164-14-701-S1.pdf]

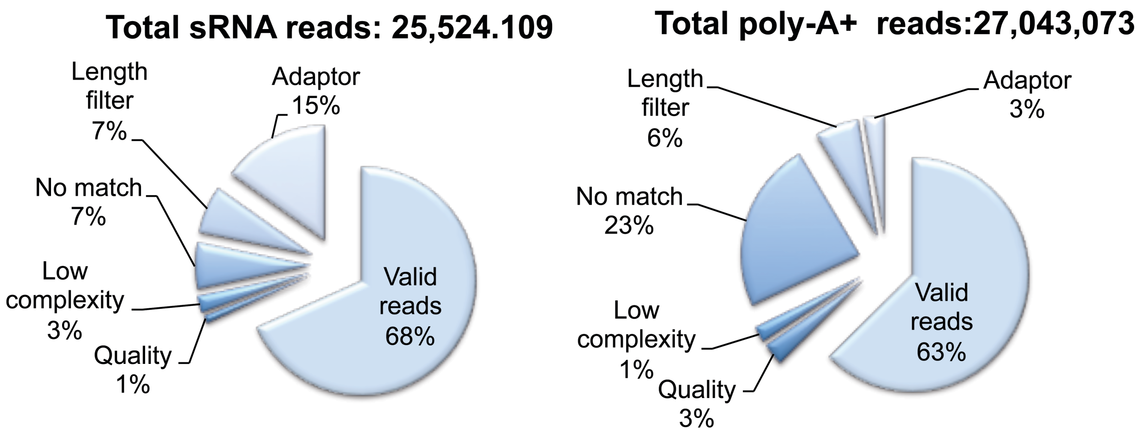

Additional file 1. Statistics of filtered sRNA and mRNA reads.

Sequences from control and treatment sRNA and poly-A+ libraries (2 biological replicates each) were filtered using FASTX and analyzed using the Novoalign program as described in Methods. We show the percentage of the total reads that did not pass the quality filter, that matched the sequencing adaptors, that had low complexity (sequences containing less than 3 different nucleotides), that were out of the size range (18-28 nt for sRNA libraries and ≥18 nt for poly-A+ libraries) and that had no perfect match with the Arabidopsis genome (TAIR v.10). Note that each pie chart represents the sum of the control and treatment libraries.
