# Supplementary material for: Integrated RNA-seq and sRNA-seq analysis identifies novel nitrate-responsive genes in Arabidopsis thaliana roots
Source: BMC Genomics. 2013 Oct 11;14:701. doi: 10.1186/1471-2164-14-701 (PMC3906980; doi:10.1186/1471-2164-14-701)
Supplement: Additional file 2 — Global profiling of Illumina-sequenced sRNA. [file 1471-2164-14-701-S2.pdf]

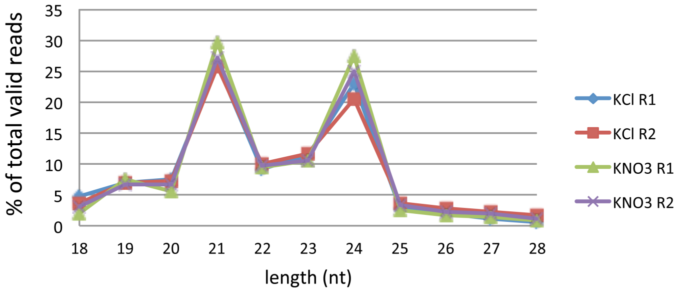

Additional file 2. Global profiling of Illumina-sequenced sRNA.

The number of sRNA reads of sizes between 18 and 28 nt that passed all the quality filters in the control and treatment libraries were counted and represented as a percentage of the total number of reads. Colors represent different sequenced libraries.
