# Supplementary material for: Integrated RNA-seq and sRNA-seq analysis identifies novel nitrate-responsive genes in Arabidopsis thaliana roots
Source: BMC Genomics. 2013 Oct 11;14:701. doi: 10.1186/1471-2164-14-701 (PMC3906980; doi:10.1186/1471-2164-14-701)
Supplement: Additional file 4 — mRNA reads that unambigoulsly match intergenic regions are located near 5’ and 3’ of annotated genes. [file 1471-2164-14-701-S4.pdf]

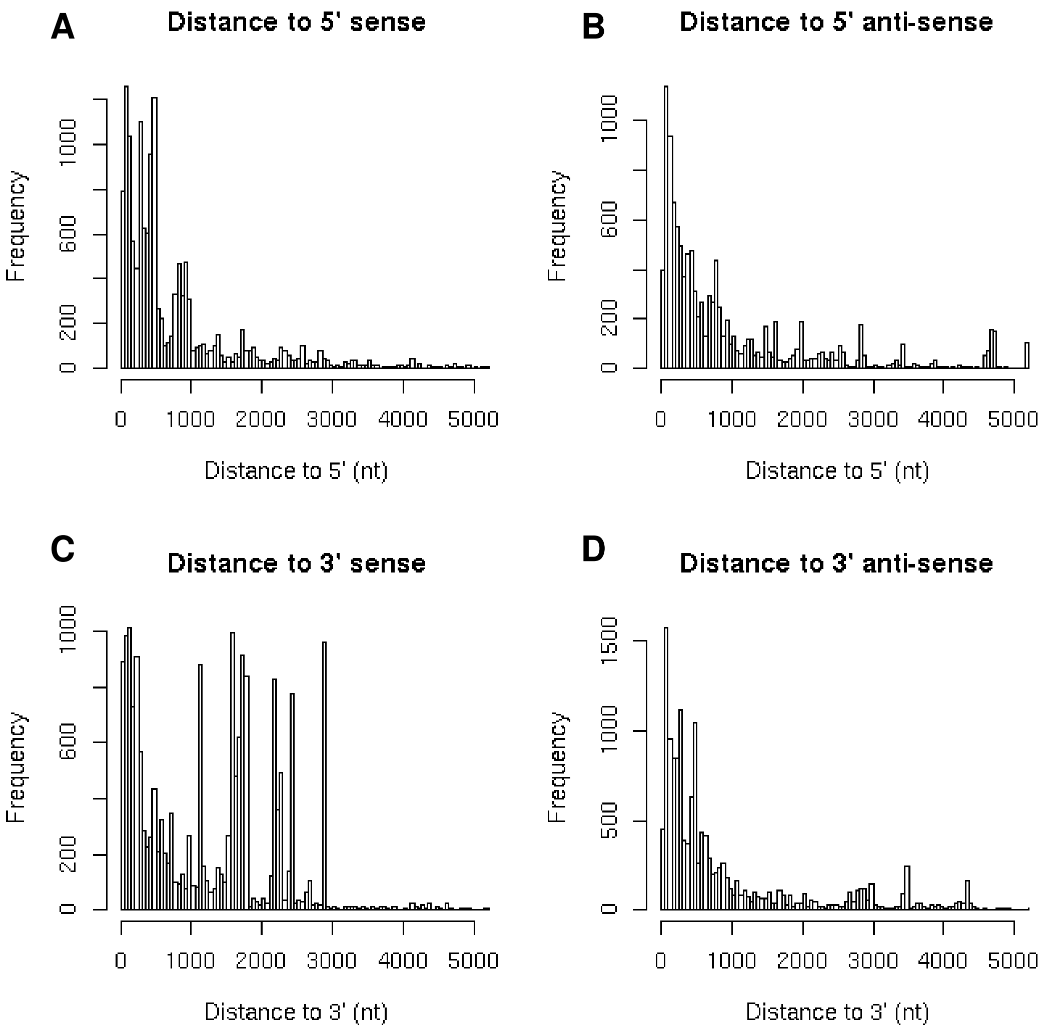

Additional file 4. mRNA reads that unambigoulsly match intergenic regions are located near 5' and 3' of annotated genes.

Reads from our poly-A+ libraries were aligned to the Arabidopsis genome. Those reads that unambiguously matched intergenic genomic regions were analyzed to determine their location relative to near annotated genes. We show reads located from 0 to 5,000 nt from the 5' and 3' of annotated genes. These reads are either sense (panels A and C) or antisense (panels B and D) to the respective genes.
