# Supplementary material for: Integrated RNA-seq and sRNA-seq analysis identifies novel nitrate-responsive genes in Arabidopsis thaliana roots
Source: BMC Genomics. 2013 Oct 11;14:701. doi: 10.1186/1471-2164-14-701 (PMC3906980; doi:10.1186/1471-2164-14-701)
Supplement: Additional file 6 — Gene expression distribution of genes represented in the ATH1 microarray in Illumina poly-A + libraries. [file 1471-2164-14-701-S6.pdf]

A

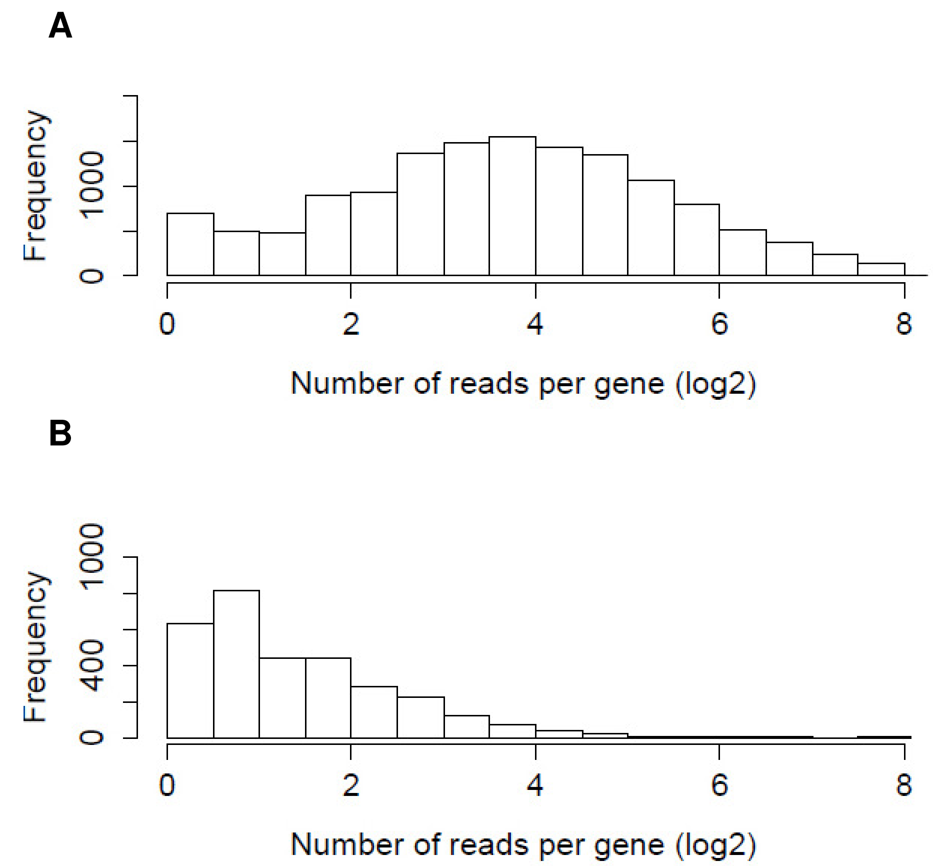

Additional file 6. Gene expression distribution of genes represented in the ATH1 microarray in Illumina poly-A+ libraries. A. Frequency and expression in Illumina libraries of genes with a present call in the ATH1 microarray. B. Frequency and expression in Illumina libraries of genes with an absent call in the ATH1 microarray.
