# Supplementary material for: Integrated RNA-seq and sRNA-seq analysis identifies novel nitrate-responsive genes in Arabidopsis thaliana roots
Source: BMC Genomics. 2013 Oct 11;14:701. doi: 10.1186/1471-2164-14-701 (PMC3906980; doi:10.1186/1471-2164-14-701)
Supplement: Additional file 7 — Correlation between nitrate-regulated genes obtained by RNA-seq and Affymetrix ATH1 microarrays depends on Average gene coverage. [file 1471-2164-14-701-S7.pdf]

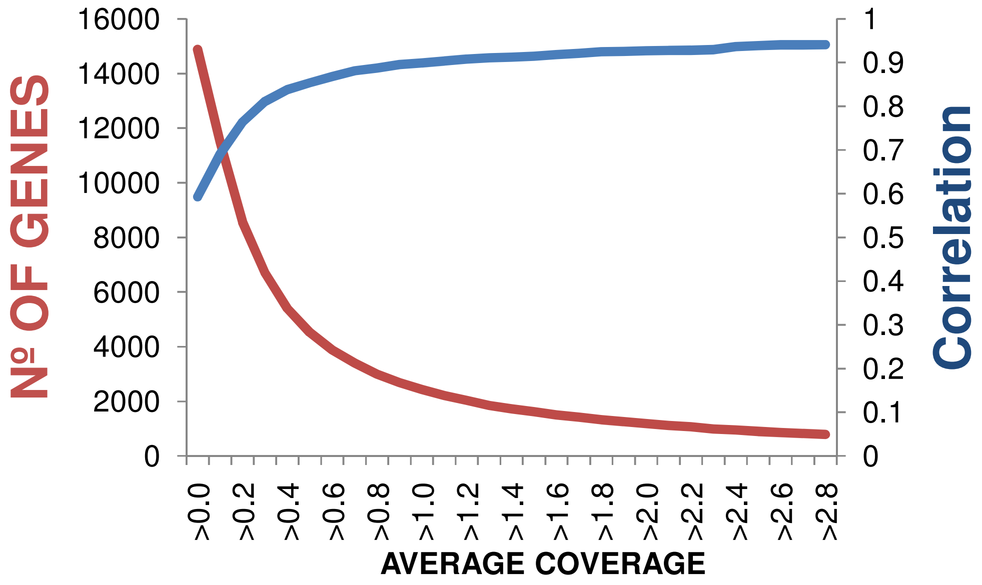

Additional file 7. Correlation between nitrate-regulated genes obtained by RNA-seq and Affymetrix ATH1 microarrays depends on Average gene coverage.

Correlation between the KNO_3/KCl ratio for RMA normalized Affymetrix gene expression and the KNO_3/KCl ratio obtained for normalized libraries at different average gene coverages (AGCs). We define AGC as the number of reads matching a gene multiplied by read length and divided by gene length. We show in blue the correlation at different AGCs and in red the number of genes at different AGCs.
