# Supplementary material for: Integrated RNA-seq and sRNA-seq analysis identifies novel nitrate-responsive genes in Arabidopsis thaliana roots
Source: BMC Genomics. 2013 Oct 11;14:701. doi: 10.1186/1471-2164-14-701 (PMC3906980; doi:10.1186/1471-2164-14-701)
Supplement: Additional file 9 — Deep sequencing of the Arabidopsis root poly-A + enriched fraction identifies new nitrate-regulated genes. [file 1471-2164-14-701-S9.pdf]

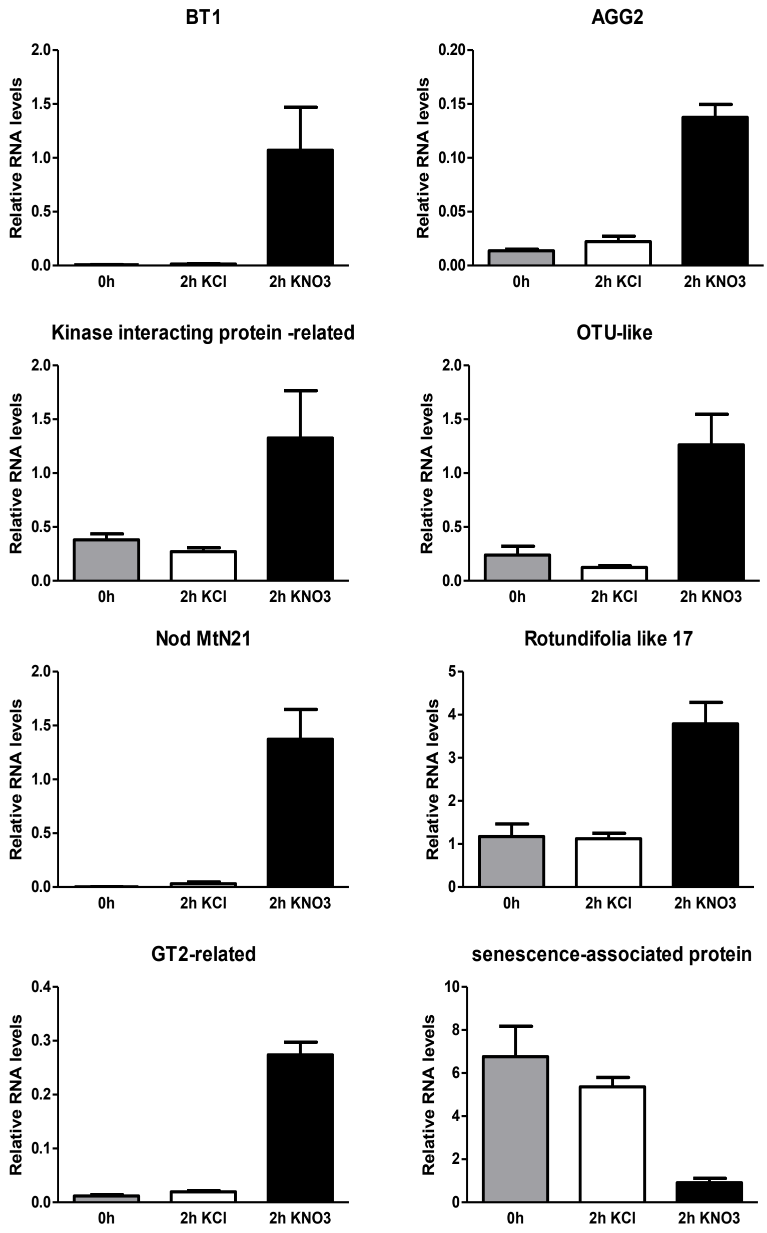

Additional file 9. Deep sequencing of the Arabidopsis root poly-A+ enriched fraction identifies new nitrate-regulated genes.

Plants were grown hydroponically for 14 days with ammonium as the sole N source and were treated with 5mM KNO_3 (black bars) or 5mM KCl (white bars) for 2 hours. Grey bar represents time 0. Root transcript levels were analyzed by RT-qPCR. We show the mean and standard error for three biological replicates. The asterisk indicates means that significantly differ between the control and treatment conditions ($P < 0.01$). BT1 (AT5G63160), AGG2 (AT3G22942), Kinase interacting protein-related (AT5G58320), OTU-like (AT5G03330), Nod MtN21 (AT1G70260), Rotundifolia like 17 (AT1G13245), GT2 related (AT2G33550), senescence associated protein (AT4G39795).
